# Evolving Rib Re-fractures in a Patient With Metal Fixators Post a Road Traffic Accident: An Unusual Hardware-Related Complication

**DOI:** 10.7759/cureus.84352

**Published:** 2025-05-18

**Authors:** Andrés David De León Murillo, Juan Carlos Varon Cotes, Fedor Andrés Spadafora Alarcón, Alejandro Alfondo Bedoya Rinaldi, Silvia Fernanda Anaya Meza, David Raúl Cerra Ortegón, Pedro Antonio Plaza Ricardo, Tatiana Paola Pérez García

**Affiliations:** 1 General Surgery Department, Universidad Libre de Barranquilla, Barranquilla, COL; 2 Thoracic Surgery, Clínica Mar Caribe, Santa Marta, COL; 3 General Physician, Universidad Libre de Barranquilla, Barranquilla, COL

**Keywords:** complication, rib fixation, rib fractures, thorax surgery, trauma

## Abstract

A rib fracture is an entity that frequently occurs after thoracic trauma. Among post-surgical complications, a rib re-fracture is a rare complication that occurs in up to 0.1% of cases.

We present the case of a 52-year-old woman who was admitted to the emergency room for polytrauma and who received surgical treatment for rib fractures. One month after discharge, she was readmitted for pain, and displacement of fixed rib fractures was found, requiring further surgical intervention. Finally, the patient was discharged due to adequate clinical evolution.

## Introduction

Thoracic injuries are the third most common injuries in trauma patients, following head injuries and extremity injuries. Rib fractures are the most prevalent injuries following thoracic trauma, and they can occur over a wide spectrum of severity, ranging from a simple rib fracture to the presentation of a flail chest [1]. These injuries may be present in up to 10% of polytraumatized patients and 39% of patients with non-penetrating thoracic trauma [2]. Each year, it is estimated that there are approximately 248,000 emergency department admissions in the United States with a diagnosis of rib fracture [3].

Rib fractures are associated with significant morbidity in patients and can range from severe pain to long-term disability. In addition, the mortality rate among hospitalized patients with rib fractures can range from 10% to 22%, being higher in elderly patients [1].

The diagnosis of this condition can be made through a physical examination; however, it is definitively confirmed by imaging, with computed tomography being the study with the highest sensitivity and specificity for this purpose [2].

The management of rib fractures can be either surgical or non-surgical, with surgical management indicated in cases of flail chest, chest wall deformity, and the presence of severe pain. Other factors taken into account in making this decision include the patient’s age, the presence of comorbidities, and the mechanism of injury [4].

Among the complications that may occur after rib fracture fixation are nonunion of the fracture, infection, bleeding, and complications related to the implant (such as irritation or failure), among others. Of these, postoperative bleeding, pain, and pneumonia are the most frequent complications; on the other hand, complications such as rib rupture and implant failure have a very low incidence, which may be less than 1% [5].

## Case presentation

A 52-year-old woman presented to the emergency department with a history characterized by polytrauma following a traffic accident (pedestrian vs. motorcycle), as a pedestrian. On admission, the patient's vital signs were as follows: temperature 37.1°C, heart rate 106 beats per minute, respiratory rate 28 breaths per minute, blood pressure 110/70 mmHg, oxygen saturation 95% on room air; Glasgow Coma Scale score 15. She had evidence of ecchymosis on the left hemithorax and crepitus upon palpation on the same side.

**Figure 1:**
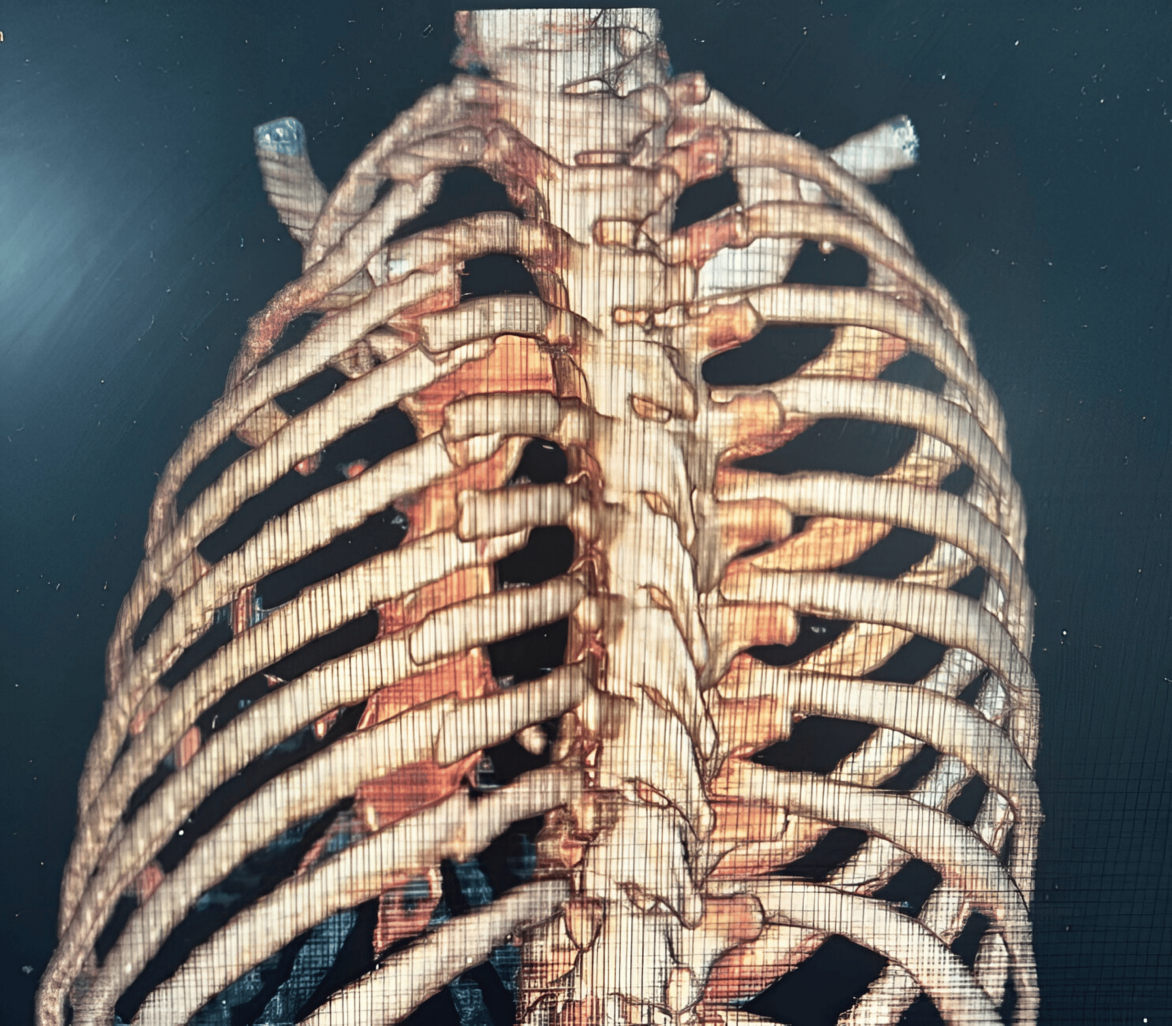
Chest computed tomography with 3D reconstruction showing evidence of displaced rib fractures from the second to the ninth rib of the left hemithorax in the postero-anterior view

**Figure 2:**
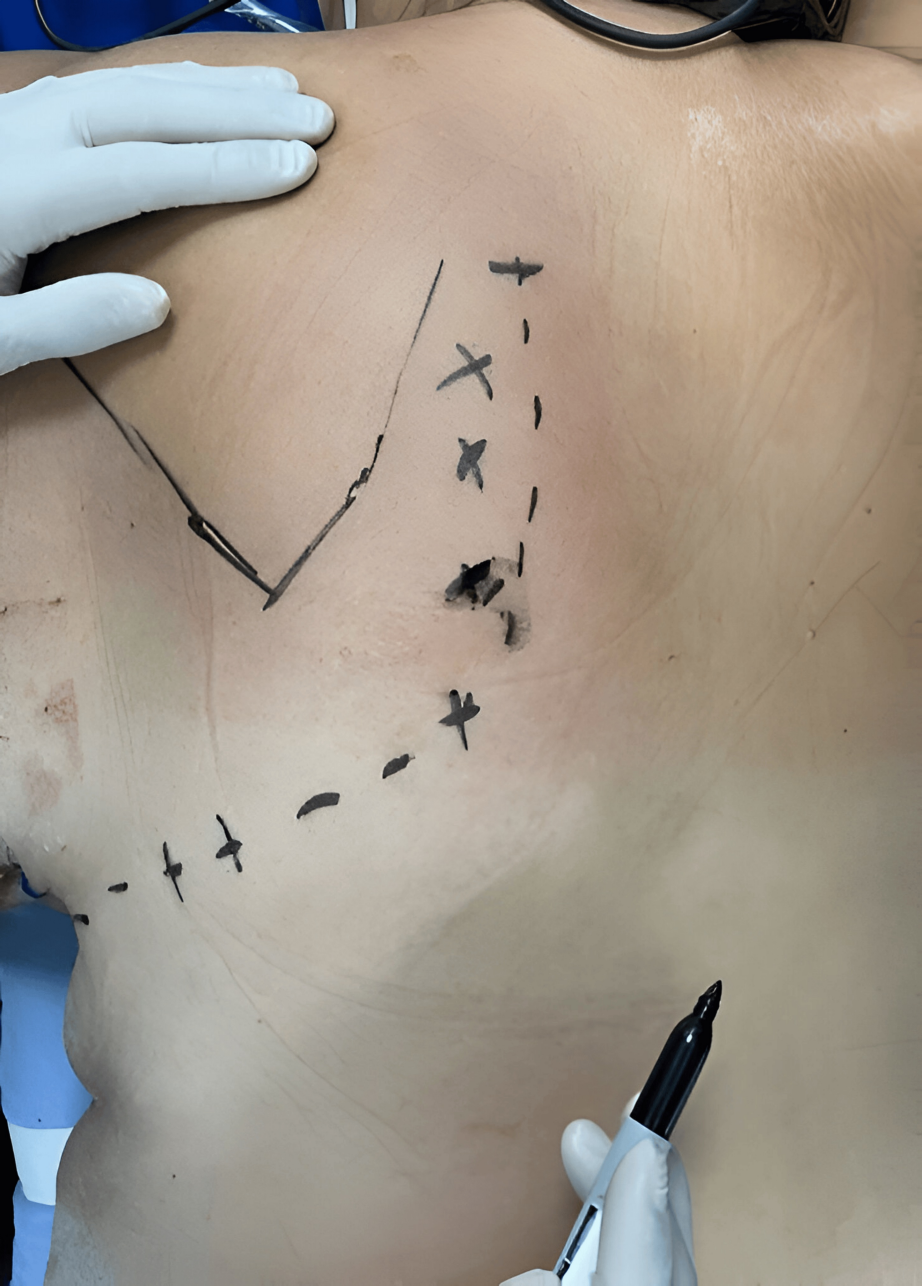
Incision marking done in the prone position

As part of the workup, a computed tomography scan of the chest with 3D reconstruction was obtained (Figure 1), which revealed displaced fractures from the second to the ninth rib. Consequently, the patient was scheduled for chest wall reconstruction using a prosthesis. In the operating room, surgical planning was performed with the patient in the prone position (Figure 2).

**Figure 3:**
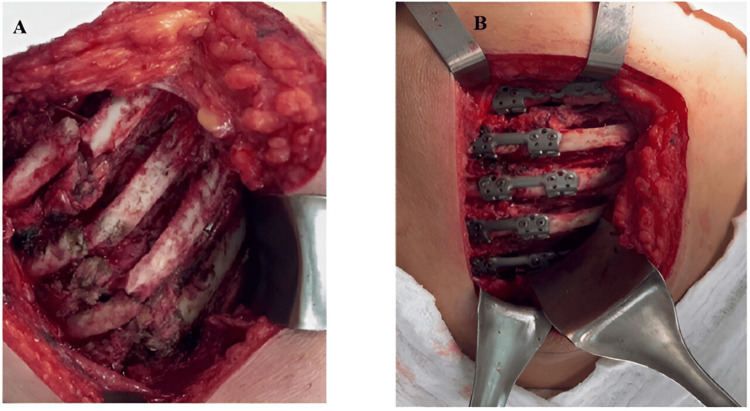
A: Exposure of rib fractures in the left hemithorax; B: Positioned rib fixation devices

Once the fracture sites were identified, rib fixation plates were placed for the reduction of the rib fractures, with fixation extending from the third rib to the eighth (Figure 3).

**Figure 4:**
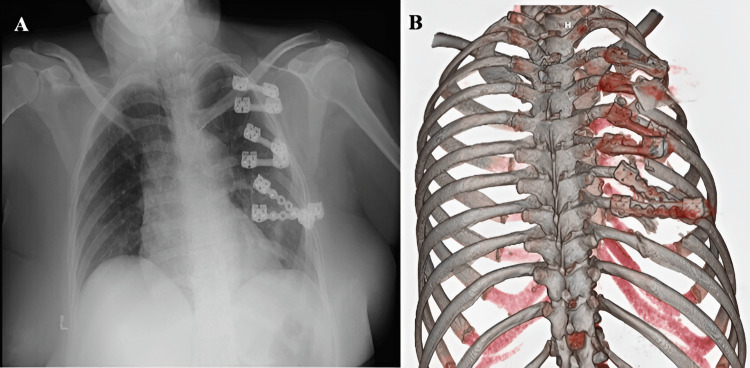
A: Chest radiograph showing evidence of displaced rib fixation devices; B: Chest computed tomography with 3D reconstruction demonstrating in greater detail the displacement of the rib fixation devices

**Figure 5:**
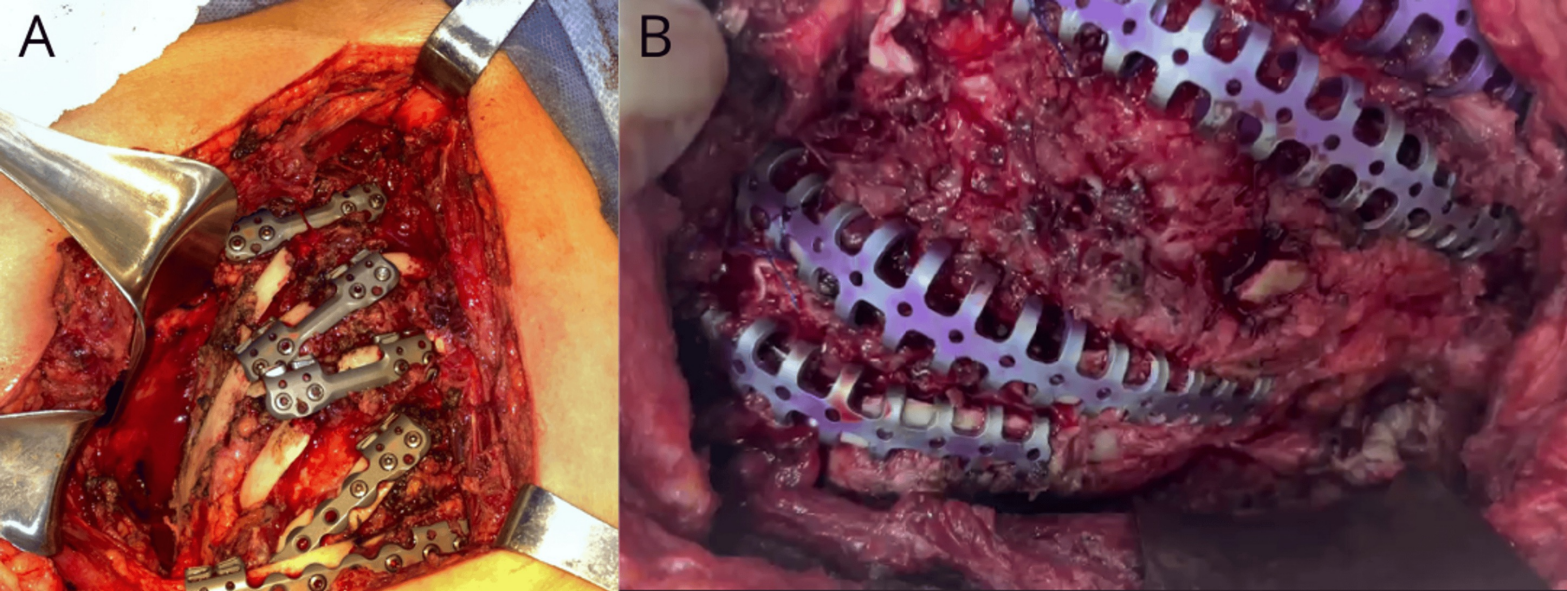
A: Displaced rib plates, with evidence of new rib fractures at both ends of the plates; B: New rib plates used for the fixation of the fourth, fifth, seventh, and eighth ribs

The patient exhibited an adequate postoperative course and was discharged five days after the procedure. She was seen in the outpatient clinic for her first postoperative follow-up 15 days later, with adequate pain management and no complications. Subsequently, the patient attended her second postoperative follow-up, one month after the procedure, where she reported a four-day history of progressively onset pain in the left posterior hemithorax. A chest radiograph was ordered, which revealed displacement of the rib fixation devices. The patient was referred to the emergency department, where a chest computed tomography scan with 3D reconstruction was performed (Figure 4), confirming the findings observed on the radiograph. Consequently, a new surgical intervention was indicated, during which displacement of all rib fixation devices was observed, compounded by the presence of new rib fractures located at both ends of each device. An attempt was made to remove the fixation screws using a tool provided by the manufacturer, without success; thus, it became necessary to perform rib resection at both the proximal and distal levels of the devices using a rib cutter. Subsequently, fixation of the fourth, fifth, seventh, and eighth ribs was performed using a fixation system different from that used in the initial surgery (Figure 5). The third and sixth ribs were left unfixed due to the absence of a sufficient rib segment for proper fixation. The patient was then transferred to the intensive care unit after the procedure was completed.

**Figure 6:**
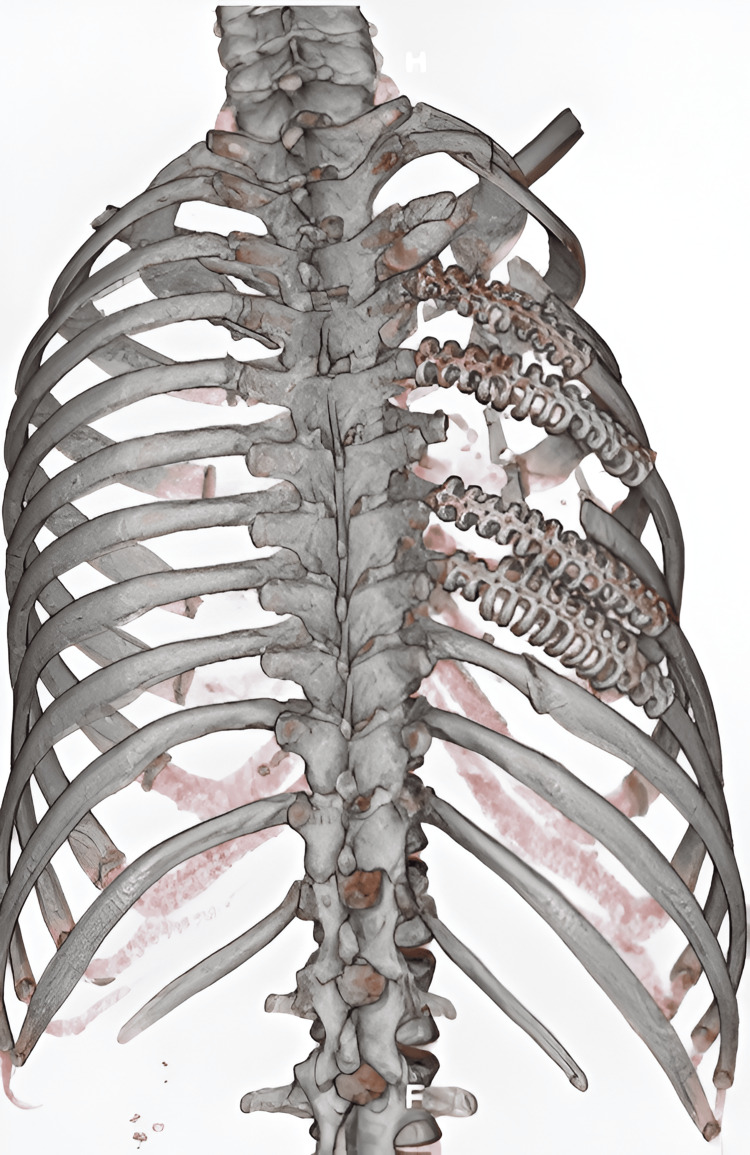
Chest computed tomography with 3D reconstruction showing evidence of properly inserted rib plates

Finally, the patient continued to exhibit an adequate postoperative course, being transferred to the inpatient ward after a 72-hour stay in the intensive care unit. She was subsequently discharged six days after the surgical intervention. The patient continues to be followed on an outpatient basis, with an adequate postoperative evolution. A new chest computed tomography scan with 3D reconstruction was requested one month after the last intervention, showing that the rib plates were properly inserted (Figure 6).

## Discussion

Rib fractures are common injuries in thoracic trauma; they can range from simple fractures to complex injuries, such as a flail chest, which can compromise the patient’s respiratory function. These fractures can involve significant complications, such as pneumonia, pneumothorax, and acute respiratory distress syndrome, especially in patients with multiple fractures or severe conditions [2,3,6]. Flail chest is a serious condition that may require immediate surgical stabilization. Rib fractures themselves are an indicator of severe injury and may be associated with a high risk of morbidity and mortality, particularly in elderly patients and those with other concomitant injuries [1,4].

The diagnosis of rib fractures is generally made through chest radiography, although computed tomography (CT) offers a more precise evaluation, especially for identifying displaced fractures or associated complications such as pneumothorax or hemothorax [6]. CT with three-dimensional reconstruction is particularly useful in surgical planning, allowing for detailed identification of the fractures and the choice of the most appropriate surgical approach [7-9]. In cases of flail chest, the diagnosis is usually clinically evident due to the instability of the chest wall, but a detailed radiological evaluation is essential to determine the extent of the fractures [7].

The treatment of rib fractures varies depending on the severity of the injury. In mild cases, conservative management is employed with adequate analgesia, including regional blocks or epidural anesthesia, and respiratory physiotherapy to prevent pulmonary complications [6]. On the other hand, the indications for surgical management include the presence of a flail chest, multiple fractures with thoracic instability, persistent pain despite conservative treatment, and significant chest wall deformity [8,9]. Although the optimal timing for surgical intervention varies, many studies suggest that early fixation, preferably within the first 24 hours, improves outcomes [5,8]. The surgical technique must be precise to avoid injury to the intercostal nerves and allow effective stabilization without compromising ventilation [8,9].

Surgical fixation of rib fractures has been shown to reduce the incidence of pulmonary complications, the need for mechanical ventilation, and the length of hospital stay [8,9].

Surgical stabilization of rib fractures can be performed using various techniques such as metal plates, absorbable plates, or intramedullary fixation. Metal plates have become the standard due to their greater stability and lower risk of complications such as nerve irritation [8,9].

The complications associated with rib fracture surgery include infections, nonunion, and implant-related issues such as irritation and displacement [5].

Postoperative follow-up should focus on monitoring respiratory complications, eradicating infections, and evaluating bone healing [5,8]. In some cases, the implants may need to be removed if they cause irritation or prove ineffective in the long term [5,9].

Managing rib fractures requires a comprehensive approach that combines conservative treatment with adequate analgesia and respiratory physiotherapy with surgical intervention when necessary. Surgical stabilization has been shown to significantly reduce pulmonary complications and hospital stay, but selecting the appropriate patients is crucial to maximizing the benefits [6,7]. Despite advances in rib fracture surgery, respiratory complications remain one of the primary concerns, underscoring the importance of a multidisciplinary approach and rigorous follow-up to improve long-term outcomes [10].

## Conclusions

Surgical fixation can be considered a safe procedure with a considerably low complication risk and satisfactory long-term outcomes, with surgery and implant-related complications in approximately 10% of patients. However, the clinically most relevant complications, such as infections, occur infrequently, and the number of complications requiring immediate (surgical) treatment is low.

Rib re-fractures as a complication of previous rib fixation are a very rare entity that, although described in recent studies with an incidence of less than 1%, cannot be completely ruled out in patients undergoing rib fixation who experience a complicated postoperative course. In this case, the complication is presented, and through the patient’s clinical presentation and the use of diagnostic imaging, the best decision for the patient can be reached.
